# Metabarcoding of bacteria and parasites in the gut of *Apodemus agrarius*

**DOI:** 10.1186/s13071-022-05608-w

**Published:** 2022-12-23

**Authors:** Soo Lim Kim, Jun Ho Choi, Myung-hee Yi, Seogwon Lee, Myungjun Kim, Singeun Oh, In-Yong Lee, Bo-Young Jeon, Tai-Soon Yong, Ju Yeong Kim

**Affiliations:** 1 Department of Environmental Medical Biology, Institute of Tropical Medicine, and Arthropods of Medical Importance Resource Bank, Yonsei University College of Medicine, Seoul, 03722 Republic of Korea; 2 Department of Biomedical Laboratory Science, College of Health Science, Yonsei University, Wonju, 26493 Republic of Korea

**Keywords:** 18S rDNA, 16S rDNA, *Apodemus agrarius*, Parasite, Microbiome

## Abstract

**Background:**

The striped field mouse *Apodemus agrarius* is a wild rodent commonly found in fields in Korea. It is a known carrier of various pathogens. Amplicon-based next-generation sequencing (NGS) targeting the 16S ribosomal RNA (rRNA) gene is the most common technique used to analyze the bacterial microbiome. Although many bacterial microbiome analyses have been attempted using feces of wild animals, only a few studies have used NGS to screen for parasites. This study aimed to rapidly detect bacterial, fungal and parasitic pathogens in the guts of *A. agrarius* using NGS-based metabarcoding analysis.

**Methods:**

We conducted 18S/16S rDNA-targeted high-throughput sequencing on cecal samples collected from *A. agrarius* (*n* = 48) trapped in May and October 2017. Taxa of protozoa, fungi, helminths and bacteria in the cecal content were then identified.

**Results:**

Among the protozoa identified, the most prevalent was *Tritrichomonas* sp*., *found in all of the cecal samples, followed by *Monocercomonas* sp. (95.8% prevalence; in 46/48 samples) and *Giardia* sp. (75% prevalence; in 36/48 samples)*.* For helminths, *Heligmosomoides* sp. was the most common, found in 85.4% (41/48) of samples, followed by *Hymenolepis* sp. (10.4%; 5/48) and *Syphacia* sp. (25%; 12/48). The 16S rRNA gene analysis showed that the microbial composition of the cecal samples changed by season (*P* = 0.005), with the linear discriminant analysis effect size showing that in the spring *Escherichia coli* and *Lactobacillus murinus* were more abundant and *Helicobacter rodentium* was less abundant. *Helicobacter japonicus* was more abundant and *Prevotella*_uc was less abundant in males. The microbial composition changed based on the *Heligmosomoides* sp. infection status (*P* = 0.019); specifically, *Lactobacillus gasseri* and *Lactobacillus intestinalis* were more abundant in the *Heligmosomoides* sp.*-*positive group than in the *Heligmosomoides* sp.*-*negative group.

**Conclusions:**

This study demonstrated that bacterial abundance changed based on the season and specific parasitic infection status of the trapped mice. These results highlight the advantages of NGS technology in monitoring zoonotic disease reservoirs.

**Graphical Abstract:**

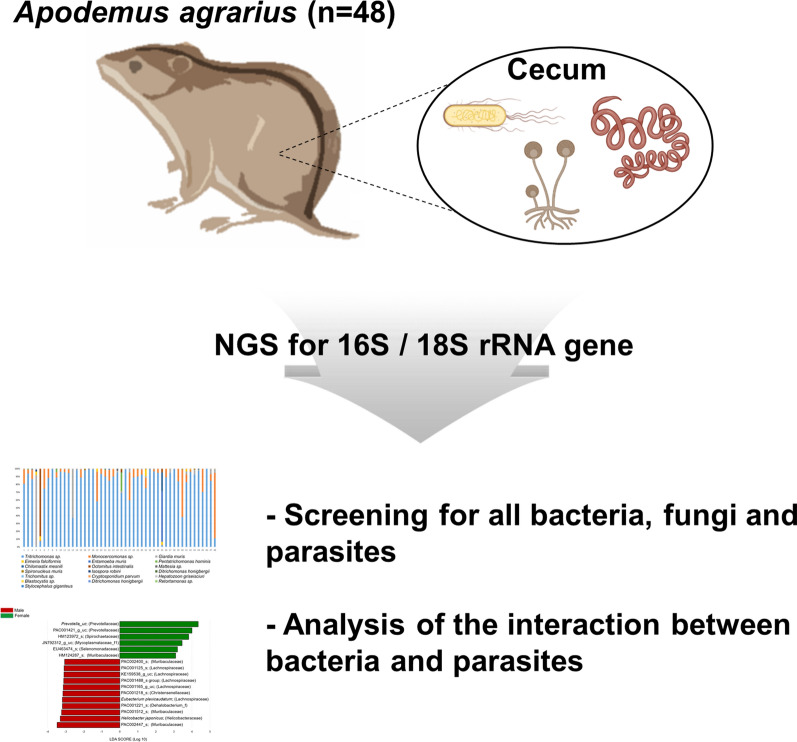

**Supplementary Information:**

The online version contains supplementary material available at 10.1186/s13071-022-05608-w.

## Background

Zoonotic diseases are commonly transmitted by wild animals around the world and can spread rapidly [1, 2]. Zoonotic diseases are caused by pathogenic organisms such as bacteria, viruses, protozoa and parasites. The emergence of novel zoonoses is generally unpredictable [3] and, therefore, it is necessary to develop a preemptive pathogen screening method for the surveillance of infected animals.

The striped field mouse, *Apodemus agrarius*, is a wild rodent that is commonly found in fields in the Republic of Korea [4]. This wild rodent can spread a great number of infectious bacteria and parasites through its feces [1, 5]. Also, rodents live in a wide variety of habitats, including agricultural regions and man-made environments [3], and they have high reproductive rates, which is a well-known characteristic of *r*-selected species. Owing to these specific characteristics, wild rodents are considered to be one of the most dangerous reservoirs of infectious organisms among wild animals.

Molecular, serological and microscopic methods, such as the PCR and enzyme-linked immunosorbent assay (ELISA), have been used to detect pathogens in wild rodent samples. For example, *Orientia tsutsugamushi*, *Anaplasma phagocytophilum* and *Leptospira interrogans* were identified in the spleens and the blood of striped field mice [6]. In addition, zoonotic helminths, such as *Hymenolepis diminuta*, were identified using light microscopy [7–10]. However, such conventional methods have limitations when screening a large number of samples for a variety of pathogens.

Recent studies have analyzed the microbiomes of feces of wild animals using next-generation sequencing (NGS) for a more integrated and rapid screening approach [11–13]. Amplicon-based NGS targeting the 16S rRNA gene is currently the most widely used technique to analyze the bacterial microbiome [14]. The NGS technique can be applied to detect veiled pathogens because of its untargeted nature and ability to investigate non-culturable organisms [15]. Although many bacterial microbiome analyses have been attempted using the feces of wild animals [16], only a few studies have used NGS to screen feces for parasites. [17–19]. Therefore, we decided to use NGS to detect various kinds of pathogens.

In this study, we used 18S ribosomal RNA (rRNA) gene amplicon-based NGS to screen for fungi and parasites and 16S rRNA gene amplicon-based NGS to screen for bacteria in the gut of *A. agrarius*. To the best of our knowledge, this is the first such study to be carried out in the Republic of Korea. A number of earlier studies have revealed the interaction between parasites and host microbiota [20–23]. Interestingly, some parasites need alterations in the host microbiota to promote their successful survival and control of parasite numbers [24, 25]. The host microbiota has also been shown to function as a resistance factor for parasitic infection [26]. Hence, we compared differences in the microbial composition of hosts based on their parasite infection status. In addition, seasonal variation could affect the host’s food availability, resulting in microbial differences and affecting the chance of infection of a parasite [27, 28]. Thus, we also compared the microbial composition based on seasonality. Cecal contents were used because these contain assorted organisms, including pathogens, and are appropriate study material for analyzing the interaction between parasites and the bacterial microbiome.

## Methods

### Sample collection

In total, 48 striped field mice (*A. agrarius*) were captured using Sherman Live Traps (H. B. Sherman Traps, Inc., Tallahassee, FL, USA) from Gangneung and Wonju, Gangwon-do, Korea in May and October 2017. Detailed information on the captured wild rodents used in this study is included in Additional file 1: Table S1. Traps were opened after 24 h; all mice were alive when the traps were opened and subsequently euthanized on the same day using a CO_2_ chamber. They were immediately dissected, and the ceca were collected and stored at – 70 °C until use. At 6 months after collection, the cecal contents were extracted from the cecal lumen using disposable sterile bacterial spreaders. Cecal DNA was extracted using the Fast DNA SPIN Kit for Soil (MP Biomedicals, Carlsbad, CA, USA) according to the manufacturer’s protocol. The DNA samples were stored at − 80 °C until needed.

### Illumina sequencing

For the eukaryotic microbiome study, the 18S rRNA gene V9 region was amplified by PCR using the primers 1391f (5′-TCGTCGGCAGCGTCAGATG TGTATAAGAGACAG GTACACACCGCCCGTC-3′) and EukBr (5′-GTCTCGTGGGCTCGGAGATGTGTATAAGAGACAGTGATC CTTCTGCAGGTTCACCTAC-3′) [29]. For the bacterial microbiome study, the 16S rRNA gene V4 region was amplified by PCR using the primers 515F (5′-TCGTCGGCAGCGTCAGATGTGTATAAGAGACAGGTGCCAGCMGCCGCGGTAA-3′) and 806R (5′-GTCTCGTGGGCTCGGAGATGTGTATAAGAGACAGGGACTACHVGGGTWTCTAAT-3′) [30]. The KAPA HiFi HotStart ReadyMix (Roche Sequencing Solutions, Pleasanton, CA, USA) was used for PCR amplification, which was performed as follows: one cycle at 95 °C for 5 min; 25 cycles of 98 °C for 30 s, 55 °C for 30 s, 72 °C for 30 s; and a final cycle of 72 °C for 5 min. AMPure XP (Beckman Coulter, Brea, CA, USA) was used for DNA purification. A limited cycling (8 cycles) amplification step was performed to add multiplexing indices and Illumina sequencing adapters. Mixed amplicons were pooled and sequenced on an Illumina iSeq 100 sequencing system using the Illumina iSeq™ 100 i1 Reagent v2 kit (Illumina Inc., San Diego, CA, USA) according to the manufacturer’s protocol.

### Processing and bioinformatics of iSeq100 data

Geneious Prime® 2022.0.2 (Biomatters Ltd., Auckland, New Zealand) was used to process and assemble raw 18S V9 reads as previously described [31, 32]. Briefly, low-quality sequences (< Q25) were filtered using BBDuk (v38.84). The forward and reverse reads were merged to produce a single consensus sequence using BBMerge (v38.84) using the ‘high rate’ setting. Sequences of 120–260 bp in length were sorted. The UCHIME algorithm was used to detect and remove chimeric sequences [33]. Closely related sequences were clustered into separate operational taxonomic units (OTUs) using de novo assembly and the default setting, which is a “Minimum Overlap Identity” of 98%. To create a sequence classifier database, the OTUs were aligned via sequence clustering using the Basic Local Alignment Search Tool (BLAST) and the NCBI “nt” GenBank database (November 2021 version). Then, the full sequences of BLAST hits from the NCBI were downloaded, and only the regions of the BLAST hits were extracted in order to create the sequence classifier database. The Geneious Sequence Classifier plugin was used to classify the merged amplicon dataset, using the created sequence classifier database. The ‘very high sensitivity/slow’ mode was used, with a minimum overlap of 90 bp. The sequences in the database that showed the highest homology were selected in the final taxonomic identification result [34].

Bacterial microbiome analysis of the 16S rRNA gene sequence data was performed using EzBioCloud, a commercially available ChunLab bioinformatics cloud platform for microbiome research (https://www.ezbiocloud.net/). Bioinformatic analyses were performed as previously described [35, 36]. Briefly, raw reads were quality checked, and low-quality (< Q25) reads were filtered using Trimmomatic 0.32 [37]. Paired-end sequence data were then merged using PandaSeq [38]. Primers were then trimmed using the ChunLab in-house program (ChunLab, Inc., Seoul, Korea), which applied a similarity cut-off of 0.8. Sequences were denoised using the Mothur pre-clustering program, which merges sequences and extracts unique sequences, allowing up to two differences between sequences [38]. The EzBioCloud database (https://www.ezbiocloud.net/) [36] was used for taxonomic assignment using BLAST 2.2.22, and pairwise alignments were generated for similarity calculations [39, 40]. The UCHIME algorithm and non-chimeric 16S rRNA database from EzTaxon were used to detect chimeric sequences for reads with a best-hit similarity rate of < 97% [33] (a 97% similarity is generally used as the cut-off for species-level identification). Sequence data were then clustered using CD-Hit and UCLUST [41, 41]. All subsequent analyses were performed using EzBioCloud.

Rarefaction for the obtained OTUs was calculated using the ChunLab pipeline, in accordance with a previous protocol [42]. The reads were normalized to 8000 for diversity analyses. We computed the Shannon index [43] and performed principal coordinates analysis (PCoA) [44], permutational multivariate analysis of variance (PERMANOVA) [45] and permutational multivariate analysis of dispersion (PERMDISP) [46] based on the generalized Bray–Curtis distance. The PERMANOVA and PERMDISP tests were used to assess the differences in the microbial community structure based on various factors, including season and parasitic infection status. We used the Wilcoxon rank-sum test to test for differences in the number of OTUs and Shannon index to compare microbiome diversity between the groups separated based on season and parasite infection status. Linear discriminant analysis (LDA) effect size (LEfSe) analysis was used to identify significantly different taxa between the groups [47]. In addition, we used the theoretical framework of a previous study to investigate the impacts (synergistic, neutral or antagonistic) of parasitic co-infection on bacterial diversity change when mice were infected by multiple parasites [48].

## Results

### Eukaryotic organisms in the *A. agrarius* gut

**Fig. 1:**
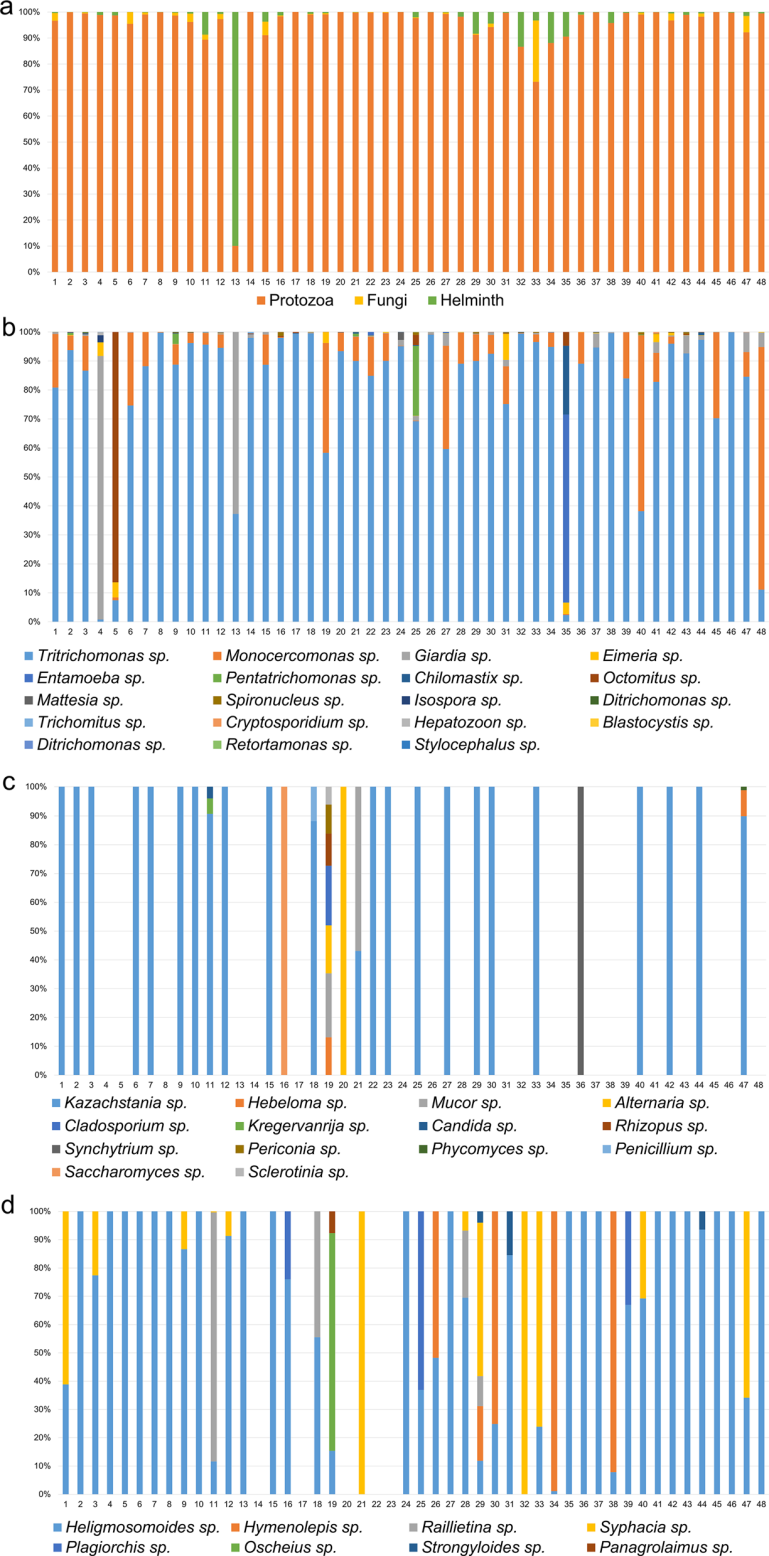
Composition of cecal eukaryotic organisms in *Apodemus agrarius* (*n* = 48 cecal samples). **a** Composition of taxa of protozoa, fungi and helminths based on 18S rRNA.** b**–**d** Taxa of protozoa (**b**), fungi (**c**) and helminths (**d**) at the species level for each sample (*n* = 48)

The average (± standard deviation [SD]) number of assigned read counts was 34,957 ± 19,899. The maximum and minimum number of reads were 88,107 and 2128, respectively. These reads included only protozoa, helminths and fungi, as the average host reads (221 ± 203) were removed before analysis. The relative abundances of fungi, protozoa and helminths in individual *A. agrarius* animals are shown in Fig. 1a. The relative abundances of protozoal taxa were greater than those of fungi and helminths in all of the *A. agrarius* samples except for one. All cecal samples were infected with *Tritrichomonas* sp. (100%, 48/48) followed in order of prevalence of infection by *Monocercomonas* sp*.* (95.8%; 46/48) and *Giardia* sp. (75%; 36/48, Fig. 1b)*.* *Isospora* sp. were found in six samples, *Cryptosporidium* sp. were found in five samples and *Blastocystis* sp*.* were found in one sample. In addition, *Entamoeba* sp., *Spironucleus* sp. and *Retortamonas* sp. were identified.

In this study, 27 of the 48 cecal samples were found to contain fungi, among which *Kazachstania* sp. was the most dominant species (Fig. 1c). *Mucor* sp. were found in two samples and *Candida* sp., *Rhizopus* sp.*, Cladosporium* sp*.* and *Periconia* sp. were found in one sample (Fig. 1c).

The relative abundance of helminth species in the cecal samples was as follows: *Heligmosomoides* sp., 85.4% (41/48); *Syphacia* sp., 25% (12/48); *Hymenolepis* sp., 10.4% (5/48); *Raillietina* sp., 8.3% (4/48); *Strongyloides* sp., 6.3% (3/48); *Plagiorchis* sp., 4.2% (2/48); *Oscheius* sp., 2.1% (1/48); and *Panagrolaimus* sp., 2.1% (1/48) (Fig. 1d). Five of 23 mice were infected with *Hymenolepis* sp. in the fall, but there were no cases of *Hymenolepis* sp. infection in the spring.

### Bacteria in the *A. agrarius* gut

**Fig. 2:**
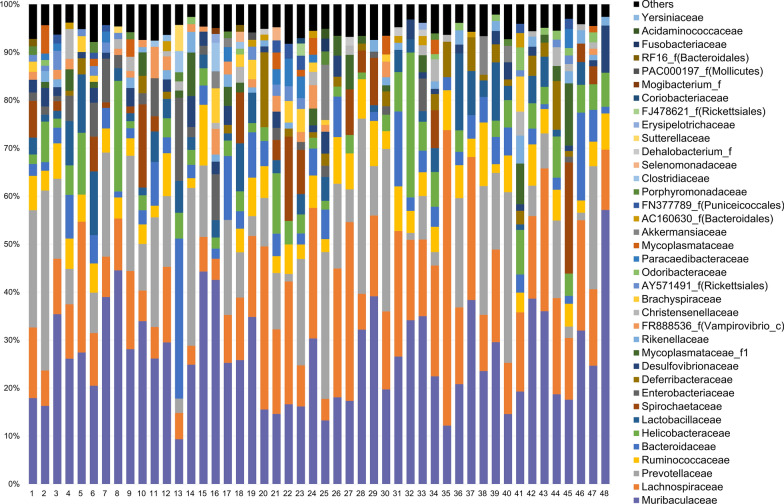
Relative abundance of bacterial taxa in the cecal microbiomes of wild *Apodemus agrarius*. Abundance was determined at the family level for each sample (*n* = 48)

High-throughput sequencing of the 16S rRNA gene of 48 cecal content samples of *A. agrarius* using the iSeq 100 system produced an average (± SD) number of assigned read counts of 27,697 ± 14,281. The relative abundances of bacterial taxa in the cecal microbiomes of individual wild rodents are shown in Fig. 2. At the family level, all samples were dominated by the presence of* Muribaculaceae* (relative abundance: 9.32–57.13%; average abundance: 26.71%), followed in order of relative abundance by* Lachnospiraceae* (relative abundance: 3.95–61.59%; average abundance: 16.83%), which was also detected in all samples. Bacterial OTUs at all the taxonomic levels are provided in Additional file 2: Table S2. *Helicobacter rodentium* and *Helicobacter aurati* were detected in 100% (48/48) and 72.9% (38/48) of samples, respectively. *Helicobacter fennelliae* was found in one sample.

### Bacterial microbiome differences based on the season

**Fig. 3:**
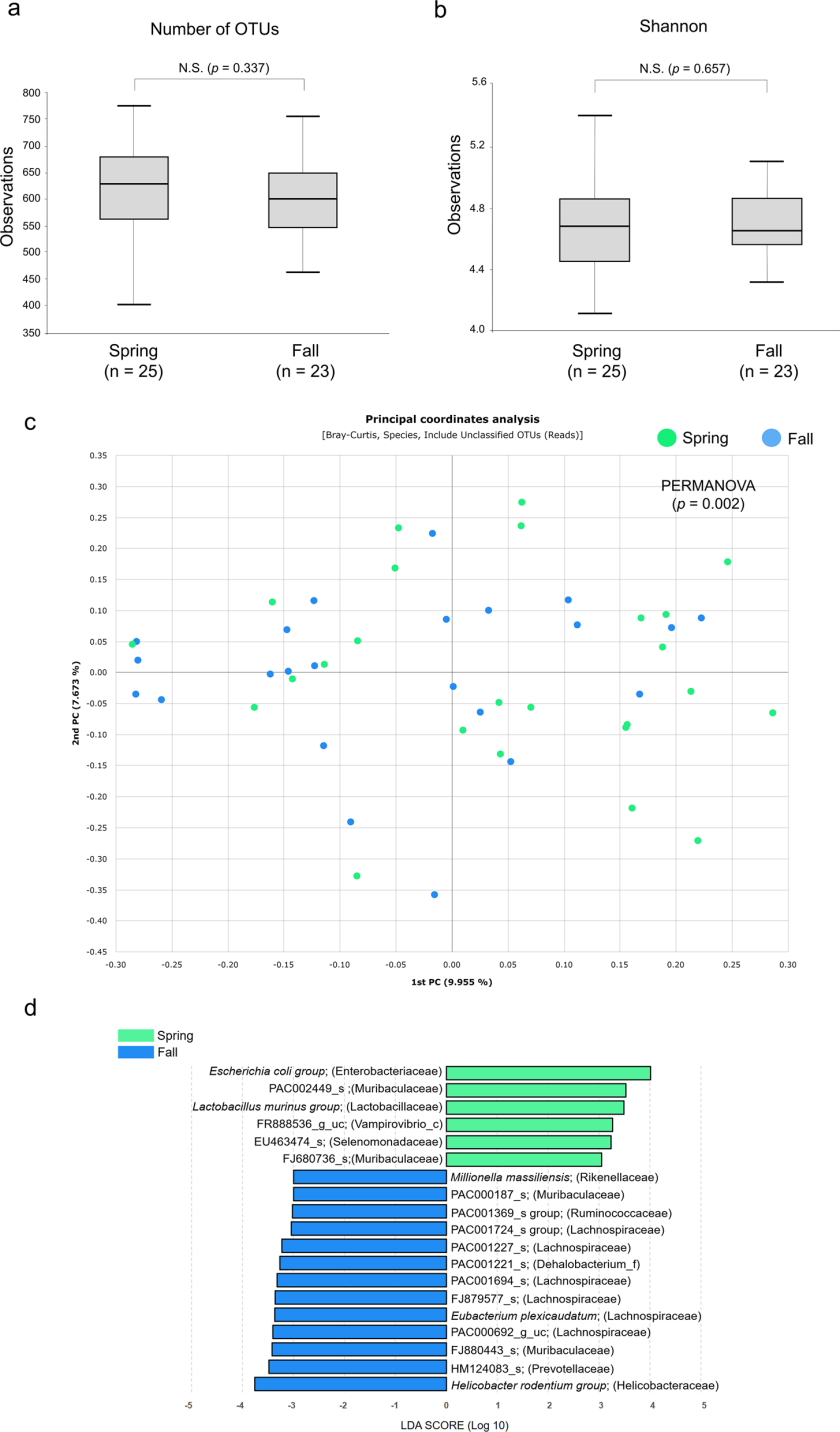
Alpha and beta diversities of the cecal microbiomes of wild *Apodemus agrarius* by collection season. **a**,** b** Box plots of number of OTUs (**a**) and Shannon index (**b**) in mice captured in the spring (*n* = 25) and those captured in the fall(*n* = 23). **c** Principal coordinates analysis representing the cecal microbiome composition. **d** Linear discriminant analysis effect size analysis of differentially abundant bacterial taxa among the two groups. Only taxa meeting a significant (> 3) linear discriminant analysis threshold are shown. OTUs, Operational taxonomic units; N.S., not significant; PERMOVA, permutational multivariate analysis of variance

The number of OTUs did not differ between the mice caught in spring (*n* = 25 mice; median OTUs: 941) and those caught in fall (*n* = 23 mice; median OTUs: 808; *P* = 0.337) (Fig. 3a). The Shannon index also did not differ between the mice caught in the spring (median Shannon index: 4.70) and those caught in the fall (median Shannon index: 4.68; *P* = 0.657 (Fig. 3b). However, the PCoA and PERMANOVA showed that the gut bacterial composition of mice caught in the spring and fall did differ significantly (PERMANOVA: *F* = 1.805, *R*^2^ = 0.042, *P* = 0.005; PERMDISP: *F* = 0.04, *R*^2^ = 0.0009, *P*= 0.83) (Fig. 3c). *Escherichia coli* (LDA score: 4.057) and *Lactobacillus murinus* (LDA score: 3.529) were more abundant in the mice collected in the spring, but *Helicobacter rodentium* (LDA score: 3.773) was less abundant in mice collected in the spring than in those collected in the fall (Fig. 3d).

### Bacterial microbiome differences based on parasitic infection status

**Fig. 4:**
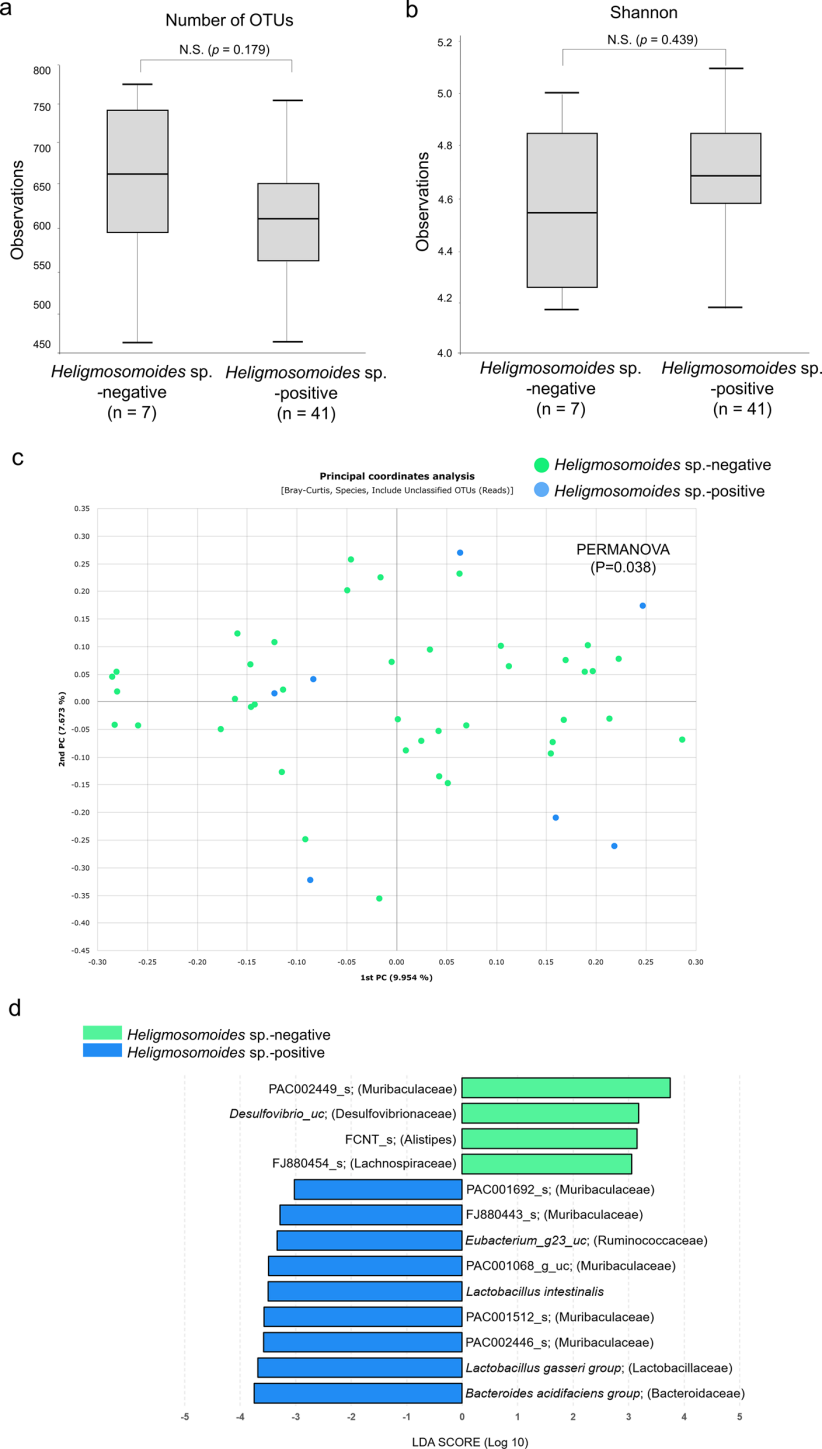
Alpha and beta diversities of the cecal microbiomes of wild *Apodemus agrarius* by *Heligmosomoides* sp*.* infection status.** a**,** b** Box plots of number of OTUs (**a**) and Shannon index (**b**) in *Heligmosomoides* sp*.*-negative (*n* = 7) and *Heligmosomoides* sp*.*-positive mice (*n* = 24). **c** Principal coordinates analysis representing the cecal microbiome composition. **d** Linear discriminant analysis effect size analysis of differentially abundant bacterial taxa among the two groups. Only taxa meeting a significant (> 3) linear discriminant analysis threshold are shown

No difference in the number of OTUs was found between *Heligmosomoides* sp.-infected mice (*n* = 41 mice; median OTUs: 582) and *Heligmosomoides* sp.-uninfected (*n* = 7 mice; median OTUs: 619) (*P* = 0.179; Fig. 4a); the Shannon index also did not differ between these two groups (median Shannon index: 4.68 [*Heligmosomoides* sp.-infected mice] vs 4.54 [*Heligmosomoides* sp.-uninfected mice]) (*P* = 0.439; Fig. 4b). PCoA and PERMANOVA showed that the gut bacterial composition was significantly different based on the *Heligmosomoides* sp. infection status (PERMANOVA: *F *= 1.408, *R*^2^ = 0.029, *P* = 0.019; PERMDISP: *F* = 0.822, *R*^2^ = 0.0176, *P* = 0.683; Fig. 4c). Interestingly, *Lactobacillus gasseri* (LDA score: 3.667) and *Lactobacillus intestinalis* (LDA score: 3.492) were more abundant in the *Heligmosomoides* sp.-positive group than in the *Heligmosomoides* sp.-negative group (Fig. 4d). We then tested the impact of *Heligmosomoides* sp. and *Giardia* sp*.* co-infection*.* Mono-infection with *Heligmosomoides* sp. did not alter the Shannon index (*P* = 0.874; Additional file 3: Fig. S1a), but the pair-wise PERMANOVA (Bray–Curtis distance) indicated significantly different microbial compositions between the *Heligmosomoides* sp. mono-infected and uninfected groups, and between the co-infected (*Heligmosomoides* sp. and *Giardia* sp*.*) and uninfected groups (*P* = 0.012 and *P* = 0.013, respectively). PERMANOVA did not indicate a significant difference between the mono-infected and co-infected groups (*P* = 0.251; Additional file 3: Fig. S1b).

## Discussion

Wild rodents are likely to play roles as vital reservoirs of zoonotic pathogens, including bacteria, parasites and fungi [49, 50]. Pathogens can be spread to humans via direct contact or through the ingestion of food and water contaminated with rodent feces [51]. In the present study, we comprehensively investigated the presence of prokaryotic and eukaryotic organisms in the gut of *A. agrarius* using metabarcoding and analyzed interactions among them.

Using the screening method described in the Methods section, i.e. the Illumina iSeq 100 system, we detected potential prokaryotic and eukaryotic pathogens. The metabarcoding method has many advantages over conventional methods, such as PCR and microscopic and culture-based screening. The metabarcoding technique can be applied when screening a large sample of a variety of pathogens at one time, thereby saving costs and decreasing the amount of labor and time required for the analysis. This technique can also be used to detect veiled pathogens because of its untargeted nature and ability to investigate non-culturable organisms, which are problematic to investigate using conventional screening methods.

Notably, this study identified various *Helicobacter* strains in feces of the collected wild *A. agrarius* (Additional file 2: Table S2). Many studies have demonstrated that wild rodents can be reservoirs of various *Helicobacter* strains [52–54]. *Helicobacter fennelliae*, which was identified in one sample in the present study, is known to cause gastroenteritis in immunocompromised humans [55]. Our results showed that *A. agrarius* is a repository of various *Helicobacter* strains, some of which may be pathogenic to humans. *Serratia marcescens*, known as an opportunistic pathogen, was also detected in 17 samples in the present study, and this species can cause severe symptoms in patients, such as sepsis, pneumonia and meningitis [56]. *Leptospira interrogans*, *O. tsutsugamushi*, and *A. phagocytophilum* are known as infectious pathogens and were previously reported to be present in the spleen, kidney and blood of *A. agrarius* at a prevalence of 4.92%, 17.6% and 19.1%, respectively; however, these species were not detected in the current study [7].

Similar to a previous study conducted in the UK, we found a lower relative abundance of *Lactobacillus* in the samples collected in the fall compared to those collected in the spring, whereas there was a higher relative abundance of *Helicobacter* in the fall [52].

Unlike bacterial community studies using the 16S rRNA gene, metabarcoding analysis targeting eukaryotic communities is still in its early stage. In the present study, we identified the infection status of parasites and fungi in the rodent gut through 18S rRNA gene amplicon sequencing. A previous study that analyzed the feces of seven *Rattus norvegicus* and two *Rattus rattus* demonstrated the strength of the metabarcoding method in comparison to microscopy [57]. In that previous study, all of the different helminths, such as *Ascaridia* and *Hymenolepis,* found using microscopy were also detected by the Illumina-based metabarcoding method [57]. In our study, we used the NCBI database as it contains a greater range of data than the SILVA database used in that previous study. For example, *Heligmosomoides* sp. was found in the NCBI database and not in the SILVA database.

The samples tested in the current study were found to contain many of the parasites reported in previous publications to be present in wild mice [50, 58–63]. In our study, we identified *Isospora* sp.*, Cryptosporidium* sp. and *Blastocystis* sp*.*, all of which might include zoonotic agents. *Cryptosporidium parvum*, for example, is a zoonotic pathogen that causes diarrhea in humans [63]. We also identified potential fungal pathogenic agents, including *Mucor* sp. and *Rhizopus* sp., which are major fungal pathogens that can cause mucormycosis in humans [64, 65]. *Cladosporium* sp., *Periconia* sp., *Candida* sp. and *Kazachstania* sp. were also found, the presence of which were previously reported in human infection cases [66–68].

Interestingly, *Hymenolepis* sp*.* was only detected in mice collected in the spring. The authors of a previous study reported that the temperature and humidity conditions during the summer and fall seasons could be advantageous for *Hymenolepis* sp*.* infection in wild rodents [60]. In the present study, we detected *Syphacia* sp. in only 25% (12/48) of samples. A previous study reported that *Syphacia* sp. could be found in 14.0% of wild rodents. Albeit rare, *Syphacia* sp. can infect humans and is a zoonotic parasite [69, 70]. *Heligmosomoides* sp. was the most prevalent infectious helminth in the present study (present in 85% of samples). A previous report suggested that intestinal helminth infections are more prevalent in *Heligmosomoides* sp.-infected wild mice than in their uninfected counterparts [61]. We detected *Raillietina* sp. in 8.3% of samples; this tapeworm was reported to have the highest prevalence of all potential zoonotic helminths infecting wild rodents in the Indochinese Peninsula [62]. We also found *Strongyloides* sp. in three samples. *Strongyloides ratti* is a skin-penetrating nematode and normally used as a laboratory model for *Strongyloides stercoralis. Oscheius* sp. and *Panagrolaimus* sp. have not been reported in wild rodents to date*. Oscheius spp.* was previously identified as an entomopathogenic nematode [71]. *Panagrolaimus spp*. is a free-living nematode that feeds on bacteria, and it has been isolated from soil [72].

*Plagiorchis* sp. was detected in three samples in the present study. Parasitic trematodes of the genus *Plagiorchis* have been reported to have zoonotic potential. *Plagiorchis muris* and *Plagiorchis elegans* have been known to cause intestinal infections in wild mice [73]. In addition, *Plagiorchis* sp. has been reported to cause intestinal infections in human patients in Japan and Korea [74]. In 2007 and 2014, *P. muris* was reported in *A. agrarius* in Korea (5.3% and 14.8%, respectively) [75, 76]. In a study carried out in the UK, 717 *P. elegans* specimens were collected from the small intestines of 27 of 117 wood mouse (*Apodemus sylvaticus*) samples [77].

There was no difference in the alpha diversity between *Heligmosomoides* sp.-infected and *Heligmosomoides* sp.-uninfected mice in the present study. This result agrees with those of Kreisinger et al. regarding the impact of helminth infections on microbial compositions [19]. In particular, we noted higher *L. gasseri* and *L. intestinalis* abundances in the *Heligmosomoides* sp.-infected group. A recent study demonstrated that *Heligmosomoides* sp. helminth infection alters the intestinal microbiota [4]. The results of other studies also confirm that the prevalence of *Lactobacillus* is increased in laboratory mice infected with various intestinal helminths [78–80].

When we analyzed the impact of *Heligmosomoides* sp. and *Giardia* sp. co-infection*,* we noted that co-infection did not cause any significantly different effects (neutral effect) compared to *Heligmosomoides* sp. mono-infection (Additional file 3: Fig. S1)*.*

Interestingly, we were able to detect *Heligmosomoides* sp. genes in the ceca despite this parasite typically residing within the small intestine. This dection is due to the sensitivity of metabarcoding analysis to detect and identify gene sequences from small amounts of parasitic cells, tissues and eggs in the ceca.

In this study, parasitic worms or eggs were not collected and identified under a microscope. In addition, since this study was conducted using the Illumina iSeq 100 system, which covers short sequence lengths, there is a limitation in the resolution of accurate identification of the parasite species. For example, the 18S V9 regions of *Heligmosomoides* sp. and *Nippostrongylus brasiliensis* differ by 1 bp although all samples had a higher identity to *Heligmosomoides* sp. Metabarcoding using various primers that target different regions of 18S rRNA gene, such as V4 and V9, may produce more accurate metabarcoding information [81].

This study did not distinguish the fungi that reside within animal guts from those that are non-residents and ingested while feeding. Further research on this topic is needed to facilitate a more precise understanding of the causes and consequences of variations in wild animal gut fungi and parasites compositions [82].

We were unable to detect blood pathogens in this study due to the nature of cecal samples. Future studies will attempt to detect such pathogens from other organ tissues.

## Conclusions

We screened bacteria, fungi, protozoa and helminths in the gut of *A. agrarius* using 16S and 18S rDNA-targeted high-throughput sequencing and identified potential zoonotic pathogens such as *Cryptosporidium* sp. and *Hymenolepis* sp. In addition, the bacterial composition was found to be changed based on the season and specific parasitic infection status of collected mice. This approach, with some improvements, will enable us to analyze a large number of samples in a high throughput manner and could be the next standard applied to investigate bacterial and parasitic infections.

## Supplementary Information

**Additional file 1: Table S1.** Date (season), location, sex, weight and *Heligmosomoides polygyrus* infection status of 48 *Apodemus agrarius.*

**Additional file 2: Table S2.** List of taxa of gut microbiome in* Apodemus agrarius*.

**Additional file 3: Figure S1.** Alpha and beta diversity of *Apodemus agrarius* cecal microbiomes based on *Heligmosomoides* sp*.* and *Giardia* sp. infection status. (**a**) Shannon index of *H. polygyrus*– *Giardia* sp. were both negative (*n *= 7), *Heligmosomoides* sp*.* single positive (*n* = 9) and *Heligmosomoides* sp*.*– *Giardia* sp. both positive mice (*n* = 32). (**b**) PERMANOVA test results representing the differences in cecal microbiome composition.

## Data Availability

Raw sequence data are available in NCBI GenBank under BioProject PRJNA864876.

## Abbreviations

LEfSe: Linear discriminant analysis effect size
NGS: Next-generation sequencing
OTU: Operational taxonomic unit
PCoA: Principal coordinates analysis
PERMOVA: Permutational multivariate analysis of variance
PERMISP: Permutational multivariate analysis of dispersion

## References

[1] Karesh WB, Dobson A, Lloyd-Smith JO, Lubroth J, Dixon MA, Bennett M (2012). Ecology of zoonoses: natural and unnatural histories. Lancet.

[2] Lloyd-Smith JO, George D, Pepin KM, Pitzer VE, Pulliam JR, Dobson AP (2009). Epidemic dynamics at the human-animal interface. Science.

[3] Han BA, Schmidt JP, Bowden SE, Drake JM (2015). Rodent reservoirs of future zoonotic diseases. Proc Natl Acad Sci USA.

[4] Sohn WM, Na BK, Song HJ, Kim CM, Nam GJ (2014). Intestinal helminthic infections in striped field mice, *Apodemus agrarius*, from two southern regions of Korea. Korean J Parasitol.

[5] Grace D, Mutua F, Ochungo P, Kruska R, Jones K, Brierley L, et al. Mapping of poverty and likely zoonoses hotspots. 2012. https://cgspace.cgiar.org/bitstream/handle/10568/21161/ZooMap_July2012_final.pdf. Accessed 20 Dec 2022.

[6] Bang MS, Kim CM, Park JW, Chung JK, Kim DM, Yun NR (2019). Prevalence of orientia tsutsugamushi, anaplasma phagocytophilum and leptospira interrogans in striped field mice in gwangju Republic of Korea. PLoS ONE.

[7] Morse SS, Mazet JA, Woolhouse M, Parrish CR, Carroll D, Karesh WB (2012). Prediction and prevention of the next pandemic zoonosis. Lancet.

[8] Yong TS, Chung KH, Ree HI (1991). Infection status of intestinal parasites of field rodents in Korea. Yonsei Rep Trop Med.

[9] Seo BS, Rim HJ, Yoon JJ, Koo BY, Hong NT (1968). Studies on the parasitic helminths of korea: iii nematodes and cestodes of rodents. Kisaengchunghak Chapchi.

[10] Lee YI, Pyeon HJ, Seo M (2013). Intestinal parasites among wild rodents in Northern Gangwon-do Korea. Korean J Parasitol.

[11] Ishida-Kuroki K, Takeshita N, Nitta Y, Chuma T, Maeda K, Shimoda H (2020). 16S rRNA gene amplicon sequence data from feces of five species of wild animals in Japan. Microbiol Resour Announc.

[12] Ishida-Kuroki K, Takeshita N, Nitta Y, Chuma T, Maeda K, Shimoda H (2020). 16S rRNA gene amplicon sequence data from feces of wild deer (*Cervus nippon*) in Japan. Microbiol Resour Announc.

[13] Bodewes R, Ruiz-Gonzalez A, Schapendonk CM, van den Brand JM, Osterhaus AD, Smits SL (2014). Viral metagenomic analysis of feces of wild small carnivores. Virol J.

[14] Knight R, Vrbanac A, Taylor BC, Aksenov A, Callewaert C, Debelius J (2018). Best practices for analysing microbiomes. Nat Rev Microbiol.

[15] Lewis E, Hudson JA, Cook N, Barnes JD, Haynes E (2020). Next-generation sequencing as a screening tool for foodborne pathogens in fresh produce. J Microbiol Method.

[16] Lavrinienko A, Hämäläinen A, Hindström R, Tukalenko E, Boratyński Z, Kivisaari K (2021). Comparable response of wild rodent gut microbiome to anthropogenic habitat contamination. Mol Ecol.

[17] Beaumelle C, Redman EM, de Rijke J, Wit J, Benabed S, Debias F (2021). Metabarcoding in two isolated populations of wild roe deer (*Capreolus capreolus*) reveals variation in gastrointestinal nematode community composition between regions and among age classes. Parasit Vectors.

[18] Aivelo T, Medlar A, Löytynoja A, Laakkonen J, Jernvall J (2018). Metabarcoding gastrointestinal nematodes in sympatric endemic and nonendemic species in Ranomafana National Park, Madagascar. Int J Primatol.

[19] Kreisinger J, Bastien G, Hauffe HC, Marchesi J, Perkins SE (2015). Interactions between multiple helminths and the gut microbiota in wild rodents. Philos Trans R Soc Lond B Biol Sci.

[20] Llinás-Caballero K, Caraballo L (2022). Helminths and bacterial microbiota: the interactions of two of humans’ “old friends”. Int J Mol Sci.

[21] Shears RK, Grencis RK (2022). Whipworm secretions and their roles in host-parasite interactions. Parasit Vectors.

[22] Wang Y, Li X, Chen X, Kulyar MF, Duan K, Li H (2022). Gut fungal microbiome responses to natural cryptosporidium infection in horses. Front Microbiol.

[23] Castañeda S, Paniz-Mondolfi A, Ramírez JD (2022). Detangling the crosstalk between Ascaris, Trichuris and gut microbiota: what´s next?. Front Cell Infect Microbiol.

[24] Hayes KS, Bancroft AJ, Goldrick M, Portsmouth C, Roberts IS, Grencis RK (2010). Exploitation of the intestinal microflora by the parasitic nematode Trichuris muris. Science.

[25] White EC, Houlden A, Bancroft AJ, Hayes KS, Goldrick M, Grencis RK (2018). Manipulation of host and parasite microbiotas: Survival strategies during chronic nematode infection. Sci Adv.

[26] Jin X, Liu Y, Wang J, Wang X, Tang B, Liu M (2022). β-Glucan-triggered *Akkermansia muciniphila* expansion facilitates the expulsion of intestinal helminth via TLR2 in mice. Carbohydr Polym.

[27] Knutie SA (2020). Food supplementation affects gut microbiota and immunological resistance to parasites in a wild bird species. J Appl Ecol.

[28] de Winter II, Umanets A, Gort G, Nieuwland WH, van Hooft P, Heitkönig IMA (2020). Effects of seasonality and previous logging on faecal helminth-microbiota associations in wild lemurs. Sci Rep.

[29] Kim JY, Choi JH, Nam SH, Fyumagwa R, Yong TS (2022). Parasites and blood-meal hosts of the tsetse fly in Tanzania: a metagenomics study. Parasit Vectors.

[30] Kim JY, Yi MH, Mahdi AAS, Yong TS (2021). iSeq 100 for metagenomic pathogen screening in ticks. Parasit Vectors.

[31] Kearse M, Moir R, Wilson A, Stones-Havas S, Cheung M, Sturrock S (2012). Geneious basic: an integrated and extendable desktop software platform for the organization and analysis of sequence data. Bioinformatics.

[32] Albakri NN, Bouqellah NA, Shabana II (2020). A metagenomic survey of lamb’s pre- and post-weaning fecal microbiomes. J Taibah Univ Sci.

[33] Edgar RC, Haas BJ, Clemente JC, Quince C, Knight R (2011). UCHIME improves sensitivity and speed of chimera detection. Bioinformatics.

[34] Kim JY, Choi JH, Nam SH, Fyumagwa R, Yong TS (2022). Parasites and blood-meal hosts of the tsetse fly in Tanzania: a metagenomics study. Parasit Vectors.

[35] Kim JY, Yi MH, Hwang Y, Lee JY, Lee IY, Yong D (2018). 16S rRNA profiling of the *Dermatophagoides farinae* core microbiome: enterococcus and bartonella. Clin Exp Allergy.

[36] Yoon SH, Ha SM, Kwon S, Lim J, Kim Y, Seo H (2017). Introducing EzBioCloud: a taxonomically united database of 16S rRNA gene sequences and whole-genome assemblies. Int J Syst Evol Microbiol.

[37] Bolger AM, Lohse M, Usadel B (2014). Trimmomatic: a flexible trimmer for Illumina sequence data. Bioinformatics.

[38] Masella AP, Bartram AK, Truszkowski JM, Brown DG, Neufeld JD (2012). PANDAseq: paired-end assembler for Illumina sequences. BMC Bioinf.

[39] Schloss PD, Westcott SL, Ryabin T, Hall JR, Hartmann M, Hollister EB (2009). Introducing mothur: opensource, platform-independent, community supported software for describing and comparing microbial communities. Appl Environ Microbiol.

[40] Altschul SF, Gish W, Miller W, Myers EW, Lipman DJ (1990). Basic local alignment search tool. J Mol Biol.

[41] Myers EW, Miller W (1988). Optimal alignments in linear space. Comput Appl Biosci.

[42] Edgar RC (2010). Search and clustering orders of magnitude faster than BLAST. Bioinformatics.

[43] Fu L, Niu B, Zhu Z, Wu S, Li W (2012). CD-HIT: accelerated for clustering the next-generation sequencing data. Bioinformatics.

[44] Heck KL, van Belle G, Simberloff D (1975). Explicit calculation of the rarefaction diversity measurement and the determination of sufficient sample size. Ecology.

[45] Shannon CE (1948). A mathematical theory of communication. Bell Sys Techn J.

[46] Gower JC (1966). Some distance properties of latent root and vector methods used in multivariate analysis. Biometrika.

[47] Anderson MJ (2001). A new method for non-parametric multivariate analysis of variance. Austral Ecol.

[48] Anderson MJ, Ellingsen KE, McArdle BH (2006). Multivariate dispersion as a measure of beta diversity. Ecol Lett.

[49] Segata N, Izard J, Waldron L, Gevers D, Miropolsky L, Garrett WS (2011). Metagenomic biomarker discovery and explanation. Genome Biol.

[50] Schmid DW, Fackelmann G, Wasimuddin RJ, Ratovonamana YR, Montero BK, et al. A framework for testing the impact of co-infections on host gut microbiomes. Anim Microbiome. 2022;4:48.. 10.1186/s42523-022-00198-5. 10.1186/s42523-022-00198-5 PMC9361228 35945629

[51] Bird BH, Mazet JAK (2018). Detection of emerging zoonotic pathogens: an integrated one health approach. Annu Rev Anim Biosci.

[52] Lee JH, Gong S, Park YC, Kim HJ, Choi IW, Lee YH (2018). Infections of intestinal helminth at two species of field mice, *Apodemus agrarius* and *A Peninsulae*, in Gangwondo and Chungcheongnam-do Korea. Korean J Parasitol.

[53] Meerburg BG, Singleton GR, Kijlstra A (2009). Rodent-borne diseases and their risks for public health. Crit Rev Microbiol.

[54] Maurice CF, Knowles SC, Ladau J, Pollard KS, Fenton A, Pedersen AB (2015). Marked seasonal variation in the wild mouse gut microbiota. ISME J.

[55] Wasimuddin, Čížková D, Bryja J, Albrechtová J, Hauffe HC, Piálek J. High prevalence and species diversity of *Helicobacter* spp detected in wild house mice. Appl Environ Microbiol. 2012;78:8158-60. 10.1128/AEM.01989-12. 10.1128/AEM.01989-12 PMC3485938 22961895

[56] Rosshart SP, Vassallo BG, Angeletti D, Hutchinson DS, Morgan AP, Takeda K (2017). Wild mouse gut microbiota promotes host fitness and improves disease resistance. Cell.

[57] O'Rourke JL, Grehan M, Lee A (2001). Non-pylori Helicobacter species in humans. Gut.

[58] Davis JT, Foltz E, Blakemore WS (1970). A pathogen of increasing clinical importance. JAMA.

[59] Tanaka R, Hino A, Tsai IJ, Palomares-Rius JE, Yoshida A, Ogura Y (2014). Assessment of helminth biodiversity in wild rats using 18S rDNA based metagenomics. PLoS ONE.

[60] Helmy YA, Spierling NG, Schmidt S, Rosenfeld UM, Reil D, Imholt C (2018). Occurrence and distribution of *Giardia* species in wild rodents in Germany. Parasit Vectors.

[61] Perles L, Roque ALR, D'Andrea PS, Lemos ERS, Santos AF, Morales AC (2019). Genetic diversity of *Hepatozoon* spp. in rodents from Brazil. Sci Rep.

[62] Brar SK, Singla N, Singla LD (2021). Comparative comprehensive analysis on natural infections of *Hymenolepis Diminuta* and *Hymenolepis Nana* in commensal rodents. Helminthologia.

[63] Behnke JM, Eira C, Rogan M, Gilbert FS, Torres J, Miquel J (2009). Helminth species richness in wild wood mice, *Apodemus sylvaticus*, is enhanced by the presence of the intestinal nematode *Heligmosomoides polygyrus*. Parasitology.

[64] Chaisiri K, Siribat P, Ribas A, Morand S (2015). Potentially zoonotic helminthiases of murid rodents from the Indo-Chinese peninsula: impact of habitat and the risk of human infection. Vector Borne Zoonotic Dis.

[65] Feltus DC, Giddings CW, Schneck BL, Monson T, Warshauer D, McEvoy JM (2006). Evidence supporting zoonotic transmission of *Cryptosporidium* spp. in Wisconsin. J Clin Microbiol.

[66] Roden MM, Zaoutis TE, Buchanan WL, Knudsen TA, Sarkisova TA, Schaufele RL (2005). Epidemiology and outcome of zygomycosis: a review of 929 reported cases. Clin Infect Dis.

[67] Ibrahim AS, Spellberg B, Avanessian V, Fu Y, Edwards JE (2005). Rhizopus oryzae adheres to, is phagocytosed by, and damages endothelial cells in vitro. Infect Immun.

[68] Wang WY, Luo HB, Hu JQ, Hong HH (2022). Pulmonary cladosporium infection coexisting with subcutaneous corynespora cassiicola infection in a patient: a case report. World J Clin Cases.

[69] de Oliveira Santos GC, Vasconcelos CC, Lopes AJO, de Sousa Cartágenes MDS, Filho AKDB, do Nascimento FRF (2018). Candida infections and therapeutic strategies: mechanisms of action for traditional and alternative agents. Front Microbiol.

[70] Kaeuffer C, Baldacini M, Ruge T, Ruch Y, Zhu YJ, De Cian M (2022). Fungal infections caused by *Kazachstania* spp., Strasbourg, France, 2007–2020. Emerg Infect Dis.

[71] Riley WA (1919). A Mouse *Oxyurid*, *Syphacia obvelata*, as a parasite of man. J Parasitol.

[72] Mohtasebi S, Teimouri A, Mobedi I, Mohtasebi A, Abbasian H, Abbaszadeh Afshar MJ (2020). Intestinal helminthic parasites of rodents in the central region of Iran: first report of a capillariid nematode from *Dryomys nitedula*. BMC Res Notes.

[73] Fu JR, Liu QZ (2019). Evaluation and entomopathogenicity of gut bacteria associated with dauer juveniles of *Oscheius chongmingensis* (Nematoda: Rhabditidae). Microbiologyopen.

[74] Oro V, Krnjajic S, Tabakovic M, Stanojevic JS, Ilic-Stojanovic S (2020). Nematicidal activity of essential oils on a psychrophilic *Panagrolaimus* sp (Nematoda: Panagrolaimidae). Plants.

[75] Catalano S, Nadler SA, Fall CB, Marsh KJ, Léger E, Sène M (2019). Plagiorchis sp. in small mammals of Senegal and the potential emergence of a zoonotic trematodiasis. Int J Parasitol Parasites Wildl.

[76] Hong SJ, Woo HC, Chai J (1996). A human case of Plagiorchis muris (Tanabe, 1922: Digenea) infection in the Republic of Korea: freshwater fish as a possible source of infection. J Parasitol.

[77] Chai JY, Park JH, Guk SM, Kim JL, Kim HJ, Kim WH (2007). Plagiorchis muris infection in *Apodemus agrarius* from northern Gyeonggi-do (Province) near the demilitarized zone. Korean J Parasitol.

[78] Boyce K, Hide G, Craig PS, Reynolds C, Hussain M, Bodell AJ (2014). A molecular and ecological analysis of the trematode Plagiorchis elegans in the wood mouse Apodemus sylvaticus from a peraquatic ecosystem in the UK. J Helminthol.

[79] Su C, Su L, Li Y, Long SR, Chang J, Zhang W (2018). Helminth-induced alterations of the gut microbiota exacerbate bacterial colitis. Mucosal Immunol.

[80] Zaiss MM, Rapin A, Lebon L, Dubey LK, Mosconi I, Sarter K (2015). The intestinal microbiota contributes to the ability of helminths to modulate allergic inflammation. Immunity.

[81] Kim JY, Kim EM, Yi MH, Lee J, Lee S, Hwang Y (2019). Chinese liver fluke *Clonorchis sinensis* infection changes the gut microbiome and increases probiotic *Lactobacillus* in mice. Parasitol Res.

[82] Kim JY, Kim EM, Yi MH, Lee J, Lee S, Hwang Y (2018). Intestinal fluke *Metagonimus yokogawai* infection increases probiotic *Lactobacillus* in mouse cecum. Exp Parasitol.

