# Supplementary material for: Metabarcoding of bacteria and parasites in the gut of Apodemus agrarius
Source: Parasit Vectors. 2022 Dec 23;15:486. doi: 10.1186/s13071-022-05608-w (PMC9789561; doi:10.1186/s13071-022-05608-w)
Supplement: Supplementary file 1 — Additional file 1: Table S1. Date (season), location, sex, weight and Heligmosomoides polygyrus infection status of 48 Apodemus agrarius. [file 13071_2022_5608_MOESM1_ESM.docx]

**Additional file Table S1**. Date (season), location, sex, weight, and *Heligmosomoides polygyrus* infection status of 48 *Apodemus agrarius*

|  | Date | Location | Sex | Weight(g) | *H. polygyrus* infection |
| --- | --- | --- | --- | --- | --- |
| 1 | 2017-05-19 (Spring) | N 37°72'94" E128°89'81" | M | 31.65 | Positive |
| 2 | 2017-05-19 (Spring) | N 37°72'94" E128°89'81" | M | 35.6 | Positive |
| 3 | 2017-05-19 (Spring) | N 37°72'94" E128°89'81" | M | 23.7 | Positive |
| 4 | 2017-05-19 (Spring) | N 37°72'94" E128°89'81" | F | 16.93 | Positive |
| 5 | 2017-05-19 (Spring) | N 37°72'94" E128°89'81" | F | 19 | Positive |
| 6 | 2017-05-19 (Spring) | N 37°72'97" E 128°89'69" | M | 32.24 | Positive |
| 7 | 2017-05-19 (Spring) | N 37°72'97" E 128°89'69" | F | 42.23 | Positive |
| 8 | 2017-05-19 (Spring) | N 37°72'97" E 128°89'69" | F | 24.89 | Positive |
| 9 | 2017-05-19 (Spring) | N 37°72'97" E 128°89'69" | F | 24.36 | Positive |
| 10 | 2017-05-19 (Spring) | N 37°68'79" E128°91'11" | M | 38.95 | Positive |
| 11 | 2017-05-19 (Spring) | N 37°68'79" E128°91'11" | M | 27.68 | Positive |
| 12 | 2017-05-19 (Spring) | N 37°72'97" E 128°89'69" | M | 28.34 | Positive |
| 13 | 2017-05-19 (Spring) | N 37°68'99" E 128°91'41" | M | 35.93 | Positive |
| 14 | 2017-05-19 (Spring) | N 37°68'99" E 128°91'41" | M | 31.41 | Negative |
| 15 | 2017-05-19 (Spring) | N 37°68'99" E 128°91'41" | F | 37 | Positive |
| 16 | 2017-05-19 (Spring) | N 37°68'99" E 128°91'41" | F | 18.33 | Positive |
| 17 | 2017-05-19 (Spring) | N 37°68'99" E 128°91'41" | M | 22.82 | Negative |
| 18 | 2017-05-19 (Spring) | N 37°72'97" E 128°89'69" | M | 42 | Positive |
| 19 | 2017-05-15 (Spring) | N 37°26'94" E 127°90'41" | M | 23.13 | Positive |
| 20 | 2017-05-15 (Spring) | N 37°26'94" E 127°90'41" | F | 23.32 | Negative |
| 21 | 2017-05-15 (Spring) | N 37°26'94" E 127°90'41" | M | 51.64 | Negative |
| 22 | 2017-05-15 (Spring) | N 37°26'94" E 127°90'41" | F | 25.49 | Negative |
| 23 | 2017-05-15 (Spring) | N 37°26'94" E 127°90'41" | M | 40.44 | Negative |
| 24 | 2017-05-15 (Spring) | N 37°26'94" E 127°90'41" | F | 25.5 | Positive |
| 25 | 2017-05-15 (Spring) | N 37°26'94" E 127°90'41" | M | 37.41 | Positive |
| 26 | 2017-10-17 (Fall) | N 37°68'79" E128°91'11" | M | 22.87 | Positive |
| 27 | 2017-10-17 (Fall) | N 37°68'79" E128°91'11" | F | 37.38 | Positive |
| 28 | 2017-10-17 (Fall) | N 37°68'79" E128°91'11" | M | 51.54 | Positive |
| 29 | 2017-10-17 (Fall) | N 37°68'79" E128°91'11" | M | 40.68 | Positive |
| 30 | 2017-10-17 (Fall) | N 37°68'79" E128°91'11" | F | 43.81 | Positive |
| 31 | 2017-10-17 (Fall) | N 37°72'97" E 128°89'69" | M | 16.8 | Positive |
| 32 | 2017-10-17 (Fall) | N 37°72'97" E 128°89'69" | F | 22.63 | Negative |
| 33 | 2017-10-17 (Fall) | N 37°72'97" E 128°89'69" | F | 37 | Positive |
| 34 | 2017-10-17 (Fall) | N 37°73'10" E 128°90'96" | F | 50.82 | Positive |
| 35 | 2017-10-17 (Fall) | N 37°73'10" E 128°90'96" | F | 19.87 | Positive |
| 36 | 2017-10-17 (Fall) | N 37°73'10" E 128°90'96" | M | 50.85 | Positive |
| 37 | 2017-10-17 (Fall) | N 37°73'10" E 128°90'96" | F | 16.82 | Positive |
| 38 | 2017-10-17 (Fall) | N 37°73'10" E 128°90'96" | F | 48.37 | Positive |
| 39 | 2017-10-17 (Fall) | N 37°73'10" E 128°90'96" | F | 40.97 | Positive |
| 40 | 2017-10-17 (Fall) | N 37°72'94" E128°89'81" | M | 42.18 | Positive |
| 41 | 2017-10-17 (Fall) | N 37°72'94" E128°89'81" | F | 17.6 | Positive |
| 42 | 2017-10-17 (Fall) | N 37°68'99" E 128°91'41" | F | 40.74 | Positive |
| 43 | 2017-10-17 (Fall) | N 37°68'99" E 128°91'41" | F | 20 | Positive |
| 44 | 2017-10-17 (Fall) | N 37°68'99" E 128°91'41" | M | 37.5 | Positive |
| 45 | 2017-10-17 (Fall) | N 37°68'92" E 128°91'46" | F | 28 | Positive |
| 46 | 2017-10-17 (Fall) | N 37°68'92" E 128°91'46" | F | 16.7 | Positive |
| 47 | 2017-10-17 (Fall) | N 37°68'92" E 128°91'46" | M | 19.13 | Positive |
| 48 | 2017-10-17 (Fall) | N 37°68'92" E 128°91'46" | M | 19.81 | Positive |
